# A moderated mediation model of green space exposure, mindfulness, physical activity level, and perceived stress

**DOI:** 10.3389/fpubh.2025.1674536

**Published:** 2025-10-08

**Authors:** Yuanzheng Lin, Xiujie Ma, Bin Zhao, Changxu La, Peng Zhang, Qingyuan Luo

**Affiliations:** ^1^College of Physical Education and Health Science, Yibin University, Yibin, China; ^2^School of Wushu, Chengdu Sport University, Chengdu, China; ^3^Xi’an Physical Education University, Xi’an, China; ^4^Beijing Normal University, Beijing, China

**Keywords:** urban green space, mindfulness, physical activity level, perceived stress, moderated mediation, mental health

## Abstract

**Objective:**

This study aimed to explore how urban green space exposure influences individuals’ perceived stress levels, focusing on the mediating role of mindfulness and the moderating role of physical activity.

**Methods:**

A cross-sectional survey was conducted in Chengdu, China, with 318 adult residents. Validated scales were used to assess green space exposure, mindfulness, physical activity, and perceived stress. Structural equation modeling and the PROCESS macro (Model 4 and Model 8) were used to test the mediation and moderated mediation effects.

**Results:**

Green space exposure was found to significantly reduce perceived stress both directly and indirectly via enhanced mindfulness. Mindfulness partially mediated the relationship between green space exposure and stress perception. Moreover, physical activity moderated both the green space–mindfulness and green space–stress pathways. Specifically, individuals with higher levels of physical activity experienced greater mindfulness gains from green exposure, while those with lower activity levels experienced stronger stress-relief benefits.

**Conclusion:**

This study highlights the dual cognitive and behavioral pathways through which green environments promote mental well-being. The findings provide theoretical insights for designing targeted urban health interventions that integrate green infrastructure with physical activity promotion.

## 1 Introduction

Urbanization and modern lifestyles have increasingly posed significant challenges to mental health worldwide. As cities expand and the pace of life accelerates, psychological stress has emerged as a pressing public health concern (1, 2). Chronic stress is linked to a range of mental and physical health problems, highlighting the urgent need for accessible, non-pharmacological strategies to promote psychological well-being in urban populations. In this context, urban green spaces have garnered growing attention as a promising public health resource. A growing body of evidence suggests that green space exposure is negatively associated with psychological distress, including depression (3), anxiety (4), and perceived stress (5, 6). Accordingly, “green prescriptions” have increasingly become a component of healthy city development initiatives.

Despite these insights, most existing studies have focused on the direct relationship between green space exposure and mental health outcomes (7, 8), while the underlying psychological and behavioral mechanisms remain underexplored. Clarifying how green environments influence stress-related outcomes can enhance the precision and effectiveness of nature-based health interventions. Moreover, individuals’ experiences and benefits from green spaces may vary depending on their lifestyle characteristics and behaviors. In particular, physical activity—widely recognized for its positive impact on mental health (9, 10)—may moderate the psychological benefits of green space exposure. While green spaces may encourage physical activity (11), the role of this behavior in shaping nature’s psychological effects remains insufficiently understood.

To address these gaps, the present study investigates the relationship between green space exposure and perceived stress, with a particular focus on the psychological and behavioral mechanisms involved. We propose a moderated mediation model that incorporates mindfulness as a mediating variable and physical activity as a moderating variable. Rather than examining bivariate associations alone, this model aims to offer a more nuanced understanding of how green environments alleviate stress. The findings of this study may inform more targeted and effective urban health promotion strategies.

## 2 Literature review and research hypotheses

### 2.1 The direct effect of green space exposure on perceived stress

Green space exposure refers to the frequency and extent of an individual’s contact with natural elements in urban environments, such as trees, parks, and gardens, and is typically assessed using self-reported measures that capture the frequency or ease of access to surrounding green areas (12). As a restorative environmental experience, green space exposure has been widely recognized for its capacity to alleviate psychological distress and reduce perceived stress.

According to Stress Reduction Theory (13), natural environments can evoke positive affective responses, lower physiological arousal, and facilitate recovery from stress. Drawing upon this theoretical framework, numerous empirical studies have demonstrated the psychological benefits of green space exposure (14, 15); these effects are particularly pronounced in high-density or vulnerable communities (16–18); and both proximity to and frequency of green space use have been associated with improved emotional regulation and reduced cortisol levels (19, 20). Based on these theoretical insights and empirical findings, we hypothesize that:

*H1:* Green space exposure is negatively associated with perceived stress.

### 2.2 The mediating role of mindfulness

Mindfulness refers to an individual’s ability to maintain purposeful, non-judgmental awareness of the present moment, encompassing internal sensations, emotions, and external stimuli (21). It enhances emotional regulation, cognitive flexibility, and resilience to stress. Recent studies have shown that natural environments, particularly urban green spaces, can foster mindfulness by enhancing attentional engagement, reducing rumination, and supporting reflective awareness (22–25). This relationship is theoretically supported by Attention Restoration Theory, which posits that natural environments replenish directed attention—a core component of mindfulness (26). In turn, mindfulness is strongly associated with reduced perceived stress. It enables individuals to reappraise stressful experiences and disengage from automatic negative thought patterns (27). Accordingly, the following hypothesis is proposed:

*H2:* Mindfulness mediates the relationship between green space exposure and perceived stress.

### 2.3 The moderating role of physical activity level

The positive effects of physical activity on both mental and physical health have been extensively documented (9). Individuals with differing levels of physical activity may exhibit distinct emotional and cognitive responses to environmental stimuli. Research suggests that physically active individuals may engage with green spaces in qualitatively different ways compared to their sedentary counterparts (28). Specifically, those who are more physically active tend to experience stronger and more immersive psychological responses to natural environments (29).

Physical activity has been shown to enhance bodily awareness, increase sensitivity to environmental cues, and foster richer multisensory engagement (30, 31). Within green space contexts, these effects may evoke heightened mindfulness and a stronger sense of connectedness with nature—both of which facilitate a mindful state (32). In contrast, individuals with lower levels of physical activity may interact more passively with natural environments, thereby experiencing weaker cognitive and affective benefits. Therefore, physical activity is likely to strengthen the association between green space exposure and mindfulness.

*H3:* Physical activity moderates the relationship between green space exposure and mindfulness.

Beyond cognitive effects, physical activity may also directly influence the relationship between green space exposure and perceived stress. According to the stress-buffering hypothesis, health-related behaviors such as exercise can enhance resilience to stress by improving physiological regulation, increasing endorphin production, and reducing allostatic load (33). Individuals who regularly engage in physical activity may already possess effective coping mechanisms (34), potentially amplifying or diminishing the marginal benefits of green space exposure.

On one hand, for physically active individuals who use green spaces as exercise venues, the stress-relieving benefits of nature may be reinforced. On the other hand, less active individuals—who may lack established coping strategies—could derive greater incremental benefit from exposure to natural environments due to their heightened baseline vulnerability to stress. Although empirical findings remain somewhat mixed, these patterns collectively suggest that physical activity may moderate the direct link between green space exposure and perceived stress.

*H4:* Physical activity moderates the direct relationship between green space exposure and perceived stress.

### 2.4 Hypotheses and conceptual model

**Figure 1:**
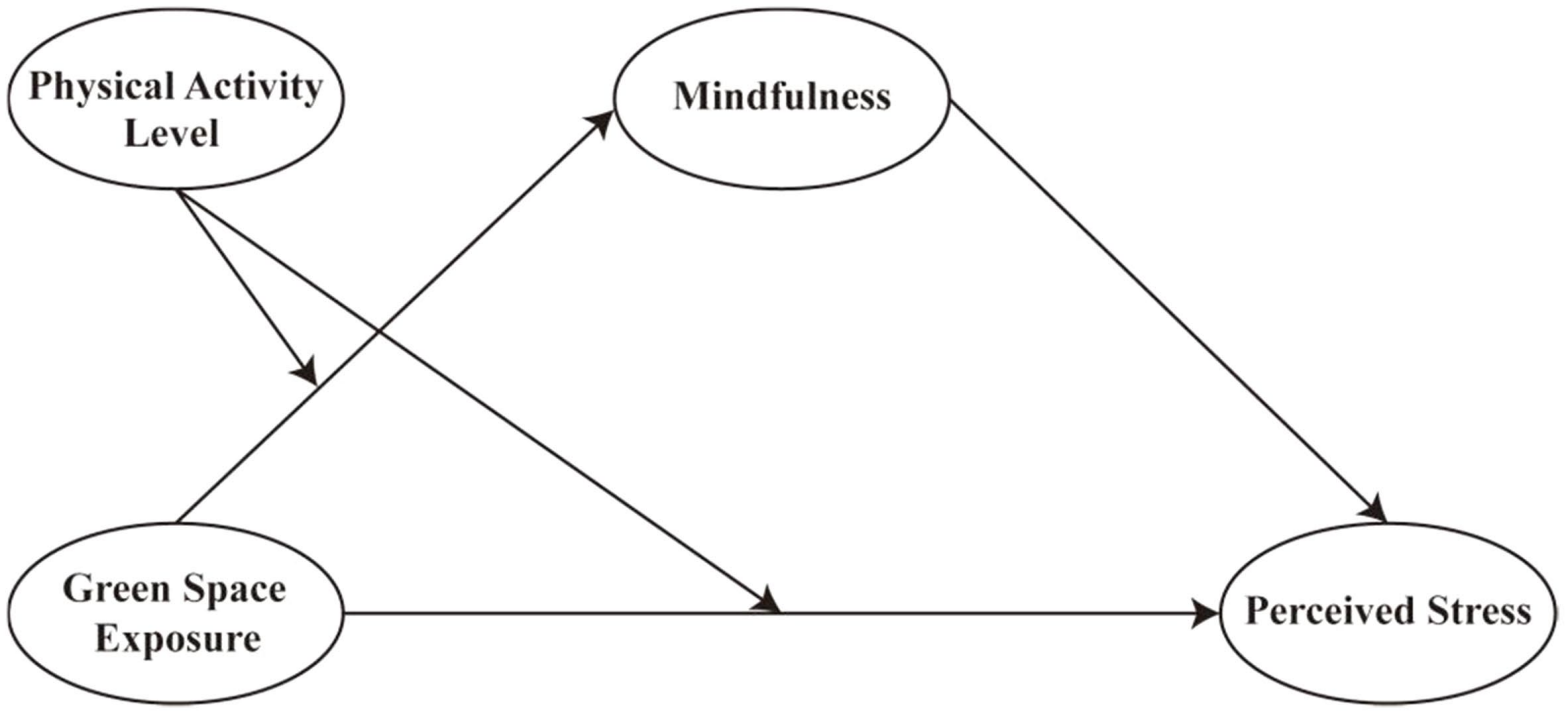
The hypothetical model.

Based on the theoretical framework and empirical evidence discussed above, this study proposes a conceptual model to examine how exposure to green spaces influences perceived stress through psychological and behavioral pathways (see Figure 1). The model posits that green space exposure is negatively associated with perceived stress both directly (H1) and indirectly through the enhancement of mindfulness (H2). Furthermore, physical activity is hypothesized to moderate the relationship between green space exposure and mindfulness (H3) as well as the direct relationship between green space exposure and perceived stress (H4).

## 3 Methods

### 3.1 Participants and procedure

This study adopted a cross-sectional survey design to examine the psychological mechanisms linking urban green space exposure to perceived stress. Data were collected in Chengdu, China, during June and July 2025—a metropolitan area known for its diverse and abundant urban green spaces. Chengdu was selected as the study site due to its strong policy emphasis on ecological livability and the continuous expansion of public green infrastructure aimed at improving residents’ well-being.

A multi-stage sampling strategy combining purposive and convenience sampling was employed. In the first stage, four urban districts were selected to represent varying levels of residential density and green space availability (e.g., dense central areas vs. low-density suburban zones). In the second stage, major public green spaces within each district—such as community parks, riverbanks, and green corridors—were identified. Trained field investigators approached individuals who appeared to be over 18 years of age and invited them to complete an anonymous questionnaire. Online recruitment was also conducted via community WeChat groups and social media, using QR codes linked to the survey.

Inclusion criteria were as follows: (1) aged 18 years or older, (2) residing in Chengdu for at least six consecutive months, and (3) having visited green spaces at least once in the past month. Participants with self-reported psychiatric conditions or severe chronic illnesses were excluded to minimize confounding effects on stress perception. After excluding incomplete responses, patterned answers, and surveys completed in under two minutes, a total of 318 valid responses were retained for analysis.

All participants provided informed consent prior to participation. This study was approved by the Research Ethics Committee of Chengdu Sport University, and all procedures complied with the Declaration of Helsinki.

### 3.2 Instruments

#### 3.2.1 Green space exposure

The measurement of green space exposure in this study was adapted from previous studies that assessed both objective proximity and subjective frequency of contact with green environments (35–37). Considering the urban context and the focus on residents’ routine experiences, this study employed a subjective exposure scale, emphasizing perceived time and frequency of contact with green spaces in everyday life. All items were rated on a 5-point Likert scale, with frequency ranging from 1 (never) to 5 (very frequently), duration from 1 (<10 min) to 5 (>1 h), and subjective exposure from 1 (strongly disagree) to 5 (strongly agree). A composite score was computed by averaging all items, with higher scores indicating higher levels of green space exposure. The internal consistency of the scale in this study was acceptable, with a Cronbach’s alpha coefficient of 0.801.

#### 3.2.2 Mindfulness

The measurement of mindfulness in this study was adapted from the Mindful Attention Awareness Scale (MAAS) developed by Brown and Ryan (38), which is one of the most widely used instruments for assessing individual differences in mindfulness in both clinical and non-clinical settings (39). The original scale comprises 15 items, focusing on the presence or absence of attention to and awareness of what is occurring in the present moment. For the purposes of this study, we selected a subset of six items that best reflected the core experiential aspects of mindfulness relevant to environmental exposure. These items emphasize individuals’ awareness of their internal states and external surroundings in daily life, particularly in relation to attentional focus and distraction. All items were rated on a five-point Likert scale ranging from 1 (strongly disagree) to 5 (strongly agree), with negatively worded items reverse-coded. In the current study, the adapted mindfulness scale demonstrated good internal consistency, with a Cronbach’s alpha coefficient of 0.872.

#### 3.2.3 Physical activity level

The measurement of physical activity level in this study was adapted from the International Physical Activity Questionnaire – Short Form (IPAQ-SF) developed by Craig et al. (40). The IPAQ-SF is a widely used and validated self-report instrument that captures the frequency and duration of vigorous physical activity, moderate physical activity, walking, and sedentary behavior over the past 7 days. Following the IPAQ scoring protocol, the weekly minutes spent in each activity category were calculated and converted into Metabolic Equivalent Task (MET) minutes per week. Total physical activity was then classified into three levels: low, moderate, and high, based on the standard IPAQ criteria. The IPAQ-SF has been shown to demonstrate acceptable reliability and validity in adult populations across multiple countries and cultural contexts (41). In the current study, the IPAQ-SF showed acceptable internal consistency with a Cronbach’s alpha coefficient of 0.812.

#### 3.2.4 Perceived stress

The measurement of perceived stress in this study was based on the Perceived Stress Scale (PSS-10) developed by Cohen, Kamarck, and Mermelstein (42), which is one of the most widely used instruments for assessing psychological stress in community and clinical populations (43). For the purposes of this study, we used the 10-item version of the PSS, which has demonstrated robust psychometric properties across diverse cultural and demographic contexts (44). All items were rated on a five-point Likert scale ranging from 1 (never) to 5 (very often), with four positively worded items reverse-coded prior to analysis. Higher scores indicate greater levels of perceived stress. In this study, the PSS-10 demonstrated excellent internal consistency, with a Cronbach’s alpha of 0.902.

### 3.3 Control variables

To account for potential confounding effects, several demographic and health-related variables were included as statistical controls based on prior research linking these factors to stress, green space exposure, and physical activity (45, 46). Specifically, the following variables were measured and controlled for in the analysis: age, gender, marital status, education level, income, occupation. These control variables were selected because they are known to influence both environmental exposure behaviors and psychological well-being.

### 3.4 Analysis

Data analysis in this study was conducted using AMOS 24.0, SPSS 26.0, and Hayes’ PROCESS macro 3.4. To assess potential common method bias (CMB), the Common Latent Factor (CLF) approach was employed. A latent method factor was added to the confirmatory factor analysis (CFA) model, allowing all observed indicators to load simultaneously on both their theoretical constructs and the CLF. Following the recommendations of Podsakoff et al. (47), model fit indices were compared between the original and the CLF-adjusted models. If the changes in GFI, CFI, TLI, and RMSEA indices are less than 0.01, then CMB is not considered a serious issue, CMB is considered not a serious concern (48). In this study, the differences in model fit indices were minimal (ΔGFI < 0.01, ΔCFI < 0.01, ΔTLI < 0.01, ΔRMSEA < 0.01), suggesting that common method bias was not a major issue.

Descriptive statistics, reliability tests, and correlation analyses were conducted using SPSS 26.0. Confirmatory factor analysis (CFA) and structural equation modeling (SEM) were performed in AMOS to evaluate both the measurement model and the structural model. Pearson correlation coefficients were used to assess the bivariate relationships among key variables.

To test the hypothesized mediating and moderating effects, the bootstrapping method (5,000 resamples) was applied using Hayes’ PROCESS macro (49). Specifically, Model 4 was used to test the mediating effect of mindfulness, and Model 8 was used to examine the moderating effect of physical activity level. In addition, a simple slope analysis was carried out to visualize the role of physical activity level in the relationship between green space exposure and mindfulness and perceived stress.

## 4 Results

### 4.1 Descriptive statistics and correlations among the Main study variables

As shown in Table 1, the final sample consisted of 318 participants (52.83% male, 47.17% female) with diverse sociodemographic backgrounds. Respondents ranged in age from 18 to over 60, with a relatively even distribution across age groups. Most participants were married (64.78%) and held at least a college-level education, with 46.86% possessing a bachelor’s degree. Common occupations included company employees, self-employed individuals, and public sector staff. Monthly income levels were primarily concentrated between 3,000 and 10,000 RMB, reflecting typical urban economic conditions.

**Table 1:** Demographic characteristics of the samples (*N* = 318).

Variable	Category	Frequency (*n*)	Percentage (%)
Gender	Male	168	52.83%
	Female	150	47.17%
Age	18–29	103	32.39%
	30–44	76	23.90%
	45–59	72	22.64%
	60 and above	67	21.07%
Marital status	Married	206	64.78%
	Unmarried	98	30.82%
	Divorced	5	1.57%
	Widowed	9	2.83%
Education level	High school or below	38	11.95%
	Junior college	52	16.35%
	Bachelor’s degree	149	46.86%
	Master’s or above	79	24.84%
Occupation	Company employee	96	30.19%
	Self-employed	69	21.70%
	Government/institutional	51	16.04%
	Student	59	18.54%
	Retired	28	8.81%
	Other	15	4.72%
Monthly income (RMB)	Under 3,000	56	17.61%
	3,001-6,000	89	27.99%
	6,001-10,000	104	32.70%
	10,000 and above	69	21.70%

Table 2 presents the means, standard deviations, and Pearson correlation coefficients among the key study variables. The mean scores ranged from 2.91 to 3.16, with standard deviations between 0.72 and 0.78, suggesting moderate central tendencies and acceptable variability. All variables were significantly correlated in the theoretically expected directions. Notably, none of the bivariate correlations exceeded the commonly accepted multicollinearity threshold of 0.85, and all variance inflation factor (VIF) values fell within acceptable limits, indicating no concerns regarding multicollinearity.

**Table 2:** Descriptive statistics and correlations among primary variables.

Variable	*M*	SD	1	2	3	4
1. Green space exposure	3.13	0.78	1			
2. Mindfulness	2.91	0.74	0.662^**^	1		
3. Physical activity level	3.16	0.73	0.622^**^	0.692^**^	1	
4. Perceived stress	2.95	0.72	−0.744^**^	−0.763^**^	−0.794^**^	1

*p < 0.05; **p < 0.01 (two-tailed).

### 4.2 The test of reliability and validity

Table 3 presents the assessment of reliability and convergent validity for the measurement model, based on the composite reliability (CR) and average variance extracted (AVE) of each latent construct. The CR values for the four core variables ranged from 0.875 to 0.939, and the AVE values ranged from 0.607 to 0.717. Following Chin (50), CR values above 0.70 and AVE values exceeding 0.50 indicate acceptable levels of internal consistency and convergent validity, respectively.

**Table 3:** Validity and reliability tests of the questionnaires.

Variable	CR	AVE
Green space exposure	0.875	0.700
Mindfulness	0.923	0.668
Physical activity level	0.883	0.717
Perceived stress	0.939	0.607

CR, composite reliability; AVE, average variance extracted.

In addition, the overall model fit was evaluated using multiple fit indices, including χ^2^/df, GFI, RFI, CFI, NFI, IFI, and RMSEA. As shown in Table 4, all fit indices exceeded the commonly recommended threshold of 0.90, with GFI = 0.925, RFI = 0.941, CFI = 0.984, NFI = 0.949, and IFI = 0.985, indicating a good model fit. The RMSEA value was 0.036, which is well below the conventional cutoff value of 0.08, suggesting a close and acceptable fit between the hypothesized model and the observed data (65).

**Table 4:** Model fit indices for the measurement model.

	χ^2^/df	*P*	GFI	RFI	CFI	NFI	IFI	RMSEA
Indices	1.408	< 0.001	0.925	0.941	0.984	0.949	0.985	0.036

GFI, goodness of fit index; RFI, relative fit index; CFI, comparative fit index; NFI, normed fit index; IFI, incremental fit index; RMSEA, root mean square error of approximation.

### 4.3 The mediation model analysis

To test the mediating role of mindfulness in the relationship between green space exposure and perceived stress, a regression-based mediation analysis was conducted using the PROCESS macro (Model 4) with 5,000 bootstrap resamples.

As shown in Table 5, green space exposure significantly predicted mindfulness (*β* = 0.627, *p* < 0.001). In the subsequent regression, both green space exposure (*β* = −0.391, *p* < 0.001) and mindfulness (*β* = −0.467, *p* < 0.001) significantly and negatively predicted perceived stress, suggesting a potential mediation effect.

**Table 5:** Regression analysis of the mediation model.

Predictors	Step 1 (Mindfulness)			Step 2 (Perceived Stress)		
	*β*	SE	*t*	*β*	SE	*t*
Green Space Exposure	0.627	0.039	15.713^***^	−0.391	0.038	−10.066^***^
Mindfulness				−0.467	0.041	−11.377^***^
*R* ^2^	0.438			0.683		
*F*	246.921^***^			340.673^***^		

*p < 0.05; **p < 0.01; ***p < 0.001.

Table 6 presents the bootstrapping results. The total effect of green space exposure on perceived stress was significant [*β* = −0.684, 95% CI (−0.752, −0.616)]. The direct effect remained significant [*β* = −0.391, 95% CI (−0.468, −0.315)], accounting for 57.16% of the total effect, thus supporting H1. The indirect effect through mindfulness was also significant [*β* = −0.293, 95% CI (−0.345, −0.242)], explaining 42.84% of the total effect, supporting H2. These findings provide empirical evidence for a partial mediation model, indicating that green space exposure alleviates perceived stress both directly and indirectly by enhancing mindfulness.

**Table 6:** Bootstrapping results of the mediation model.

	Effect	SE	95% CI	Ratio to total effect
Direct Effect	−0.391	0.038	[−0.468, −0.315]	57.16%
Indirect effect	−0.293	0.026	[−0.345, −0.242]	42.84%
Total effect	−0.684	0.034	[−0.752, −0.616]	-

### 4.4 The moderating model analysis

To examine the moderating role of physical activity in the relationships between green space exposure and mindfulness, as well as between green space exposure and perceived stress, this study employed Model 8 of the PROCESS macro for moderated regression analysis.

As shown in Table 7, when mindfulness was the dependent variable, both green space exposure (*β* = 0.373, *p* < 0.001) and physical activity (*β* = 0.435, *p* < 0.001) positively and significantly predicted mindfulness. More importantly, the interaction term between green space exposure and physical activity was also significant (*β* = 0.147, *p* < 0.001), indicating that physical activity significantly moderated this relationship.

**Table 7:** Regression analysis of the moderation model.

Variable	M: Mindfulness			Y: Perceived stress		
	*β*	SE	*t*	*β*	SE	*t*
Constant	2.866	0.031	91.565^***^	3.763	0.117	32.038^***^
Green space exposure	0.373	0.044	8.425^***^	−0.257	0.035	−7.347^***^
Mindfulness				−0.294	0.040	−7.310^***^
Physical activity level	0.435	0.048	9.052^***^	−0.420	0.038	−10.906^***^
Green space exposure × Physical activity level	0.147	0.043	3.376^***^	0.118	0.031	3.735^***^
R^2^	0.581			0.775		
*F*	145.613^***^			270.363^***^		

*p < 0.05; **p < 0.01; ***p < 0.001.

In the second model, where perceived stress was the dependent variable, green space exposure (*β* = −0.257, *p* < 0.001), mindfulness (*β* = −0.294, *p* < 0.001), and physical activity (*β* = −0.420, *p* < 0.001) all had significant negative associations with perceived stress. Additionally, the interaction between green space exposure and physical activity was significant (*β* = 0.118, p < 0.001), further confirming a moderating effect.

In summary, these results provide strong evidence for the moderating role of physical activity in both the green space–mindfulness pathway and the green space–stress pathway, thereby supporting Hypotheses 3 and 4.

**Figure 2:**
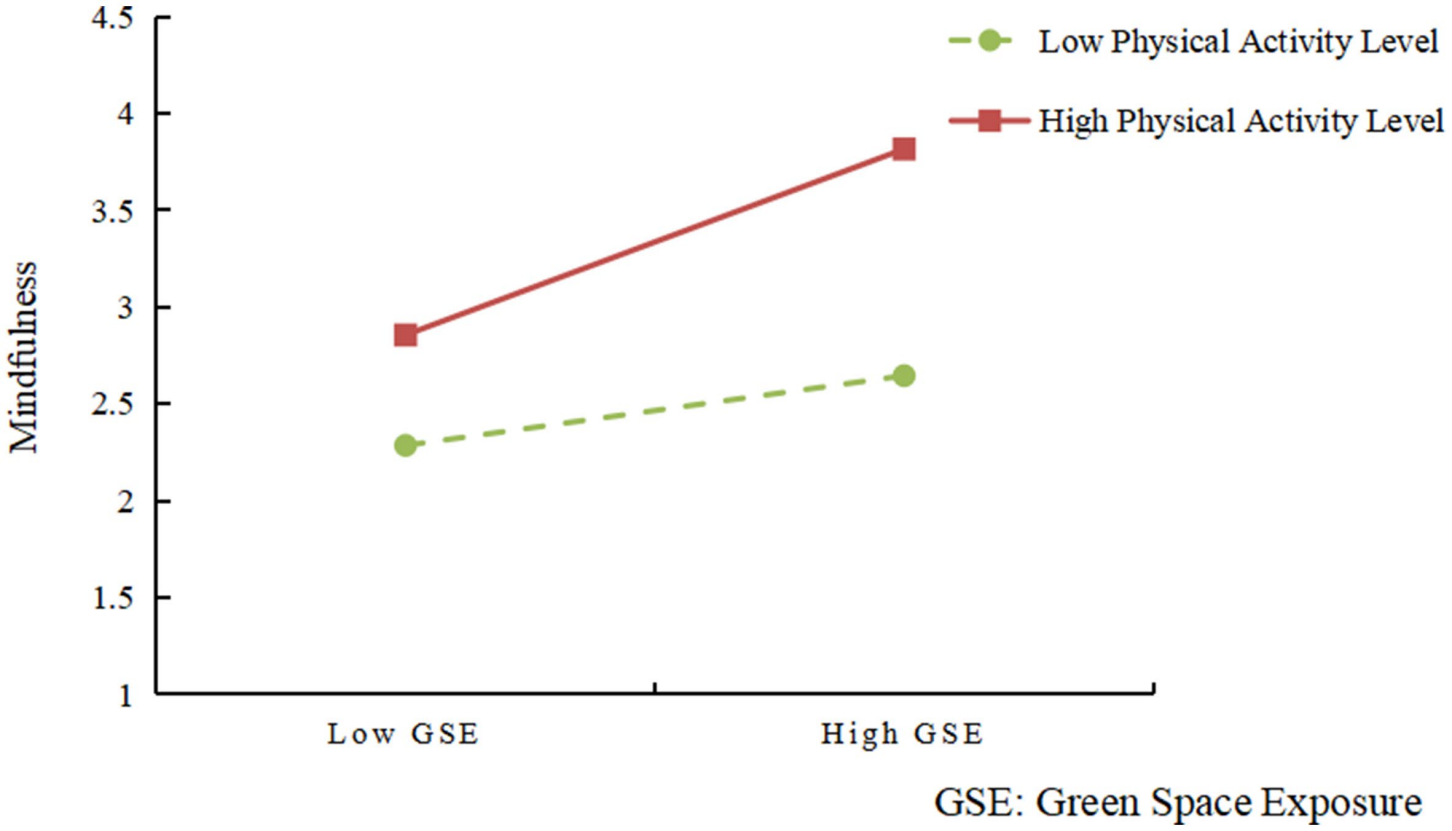
Interaction effect of green space exposure and physical activity level on mindfulness. Simple slopes analysis shows that mindfulness increases more strongly with green space exposure among individuals with high physical activity (red solid line), while the effect is weaker for those with low activity (green dashed line).

To further investigate the moderating effect of physical activity levels, a simple slopes analysis was performed. As illustrated in Figure 2, for individuals with high levels of physical activity, increased exposure to green spaces significantly enhanced mindfulness. The red solid line displays a clear upward trend, indicating that these individuals are more capable of benefiting from the heightened present-moment awareness and attentional focus fostered by green environments. In contrast, for those with low physical activity levels, the positive association between green space exposure and mindfulness was relatively weaker, as reflected by the more gradual slope of the green dashed line. These results suggest that individuals who are more physically active are better positioned to perceive and harness the psychological restorative benefits offered by natural environments.

**Figure 3:**
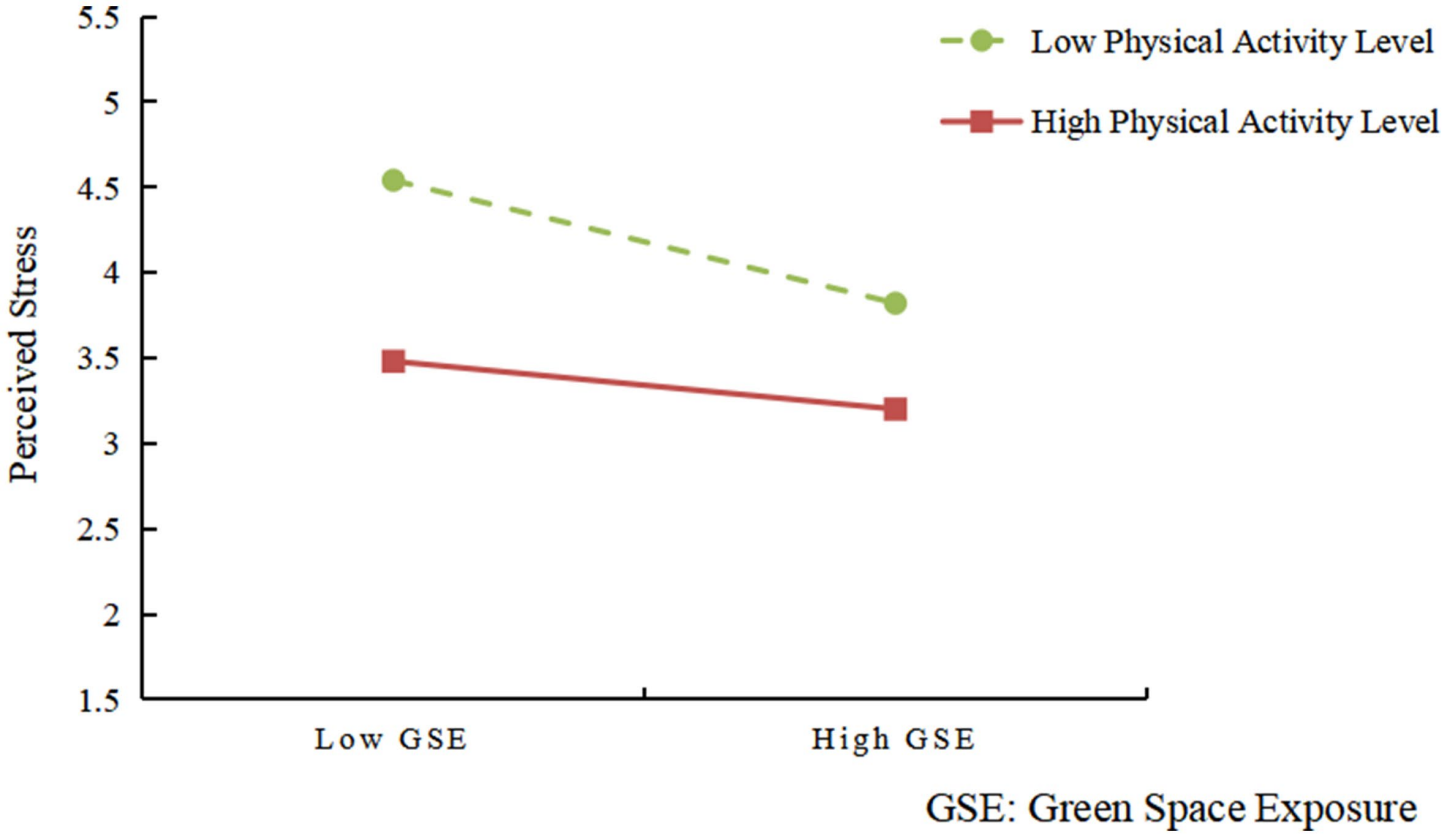
Interaction effect of green space exposure and physical activity level on perceived stress. Simple slopes analysis shows that stress decreases more sharply with green space exposure among individuals with low physical activity (green dashed line), whereas the decline is smaller among those with high activity (red solid line).

As illustrated in Figure 3, among individuals with low levels of physical activity, perceived stress decreases significantly as green space exposure increases, as indicated by the steep downward slope of the green dashed line. This suggests that the psychological benefits of green environments are more pronounced for those who are less physically active. In contrast, for individuals with high physical activity levels, the red solid line exhibits a relatively flat downward slope, indicating a weaker relationship between green space exposure and stress reduction. These findings imply that while green space exposure generally contributes to lower perceived stress, its marginal benefit is greater for those with lower physical activity levels—possibly because physically active individuals already possess enhanced stress regulation capacities.

## 5 Discussion

### 5.1 The direct effect of green space exposure

This study found that exposure to green spaces significantly and negatively predicted individuals’ perceived stress, indicating that greater contact with natural environments is associated with lower psychological stress. This result aligns with previous research, reinforcing the beneficial role of urban green spaces in promoting mental well-being (19, 51). As restorative environments, green spaces are thought to support emotional regulation, attentional recovery, and the release of negative emotions by offering quiet, safe, and aesthetically pleasing surroundings (26, 52).

Furthermore, physical attributes of green spaces—such as vegetation density, shade, noise buffering, and visual appeal—are key to facilitating psychological restoration and enhancing subjective well-being (53, 54). In high-density urban contexts characterized by chronic noise and social stress, nature exposure may serve as a psychological buffer, potentially contributing to the alleviation of accumulated mental burdens. Especially in the post-pandemic era, the role of green infrastructure in promoting public health has drawn increasing attention (55).

The present findings underscore the importance of increasing residents’ actual access to green spaces. Beyond simply expanding green area or coverage, urban planners and policymakers should emphasize spatial equity and accessibility—such as by developing pocket parks, greenways, and neighborhood-scale green interventions—to ensure that natural environments are easily reachable as part of daily routines. At the same time, the feasibility of such measures in high-density urban contexts may be constrained by factors such as land costs, long-term maintenance resources, and property rights divisions. Therefore, future strategies should involve interdisciplinary collaboration with urban planners, public health experts, and policymakers to balance health promotion goals with spatial and economic realities.

### 5.2 The mediating role of mindfulness

This study found that mindfulness significantly mediates the relationship between green space exposure and perceived stress. This finding suggests that green environments are not only associated with lower stress directly but may also indirectly relate to stress outcomes through enhanced mindfulness. In other words, natural settings may foster greater present-moment awareness and non-judgmental attention, which is associated with lower emotional reactivity to stressors.

Previous research has indicated that natural environments can promote mindfulness by enhancing attentional control and emotional regulation (56). The Attention Restoration Theory (ART) proposed by Kaplan and Kaplan (57) also supports this mechanism, suggesting that the “soft fascination” elicited by nature helps restore depleted cognitive resources, enabling more sustained attentional focus and awareness. Such effortless attentional engagement reduces cognitive fatigue and allows individuals to anchor attention to the present moment, which directly overlaps with the attentional processes central to mindfulness. From the perspective of Stress Reduction Theory (SRT), natural environments evoke positive affective responses and reduce physiological arousal, thereby creating an emotional context conducive to mindfulness.

Empirical evidence further confirms that mindfulness interventions or meditation conducted in outdoor natural settings are more effective in improving mindfulness and promoting relaxation than those conducted indoors (58, 59). At the same time, it should be acknowledged that not all studies have reported consistent results. For example, some evidence suggests that the psychological benefits of green space exposure may be stronger among higher socioeconomic status (SES) populations (60). Other studies have found that mindfulness does not always play a significant mediating role in the relationship between natural environments and mental health (61). These inconsistencies indicate that the dual-path mechanism identified in this study may be contingent upon contextual or demographic factors, and future research should further examine such boundary conditions. At the neurophysiological level, mindfulness has been linked to increased prefrontal cortex activity and reduced amygdala reactivity—patterns that are associated with lower stress perception (62). Therefore, green space exposure may be linked to mindfulness-related cognitive and emotional pathways, which in turn are associated with how individuals appraise and respond to stress.

In sum, this study not only validates mindfulness as a key psychological mechanism in the green space–stress relationship but also highlights the practical value of incorporating mindfulness training into nature-based health interventions. Future urban wellness strategies might consider integrated models such as ‘mindfulness in nature’—including practices like mindful walking or designing designated outdoor meditation zones—to offer more sustainable mental health solutions. More concretely, mindfulness can be supported through specific design elements such as establishing quiet areas for meditation, creating pathways that encourage slow and mindful walking, and incorporating landscape features that evoke ‘soft fascination’ (e.g., water features, shaded tree-lined paths, biodiversity-rich gardens). These examples provide more tangible implications for urban planning and landscape design.”

### 5.3 The moderating role of physical activity level

This study further examined the moderating role of physical activity levels in the psychological mechanisms linking green space exposure to perceived stress. The results demonstrated that physical activity significantly moderated both the direct relationship between green space exposure and perceived stress, and the indirect pathway via mindfulness. These findings suggest that individuals with different levels of physical activity exhibit distinct psychological responses to natural environments. As a behavioral trait, physical activity may function as either an amplifier or a buffer in the relationship between nature exposure and mental well-being.

On the pathway from green space exposure to mindfulness, individuals with higher physical activity levels were more likely to report stronger mindfulness associated with green space exposure. This aligns with prior studies showing that physically active individuals are more likely to experience immersion, present-focused awareness, and a heightened sense of embodiment in natural settings (63). Physical activity may enhance body awareness and attentional control, which may facilitate entry into a mindful state and potentially make the psychological benefits of green environments more accessible.

Regarding the pathway from green space exposure to perceived stress, the moderation pattern was reversed: individuals with lower physical activity levels tended to report greater reductions in perceived stress as green space exposure increased. In comparison, those with higher physical activity levels showed relatively stable stress levels, regardless of green space exposure. This may be due to the fact that physically active individuals already possess stronger coping mechanisms and psychological resilience, reducing their reliance on external restorative environments. Conversely, less active individuals may lack effective emotion regulation strategies and therefore benefit more from the stress-reducing effects of natural exposure (64).

These findings highlight the importance of accounting for behavioral differences in designing nature-based health interventions. Urban planning should integrate high-quality green infrastructure with initiatives that promote physical activity—such as greenways, exercise facilities, and signage systems—to maximize the mental health benefits of green spaces. Future studies could explore how specific dimensions of physical activity, such as type (aerobic vs. non-aerobic) and frequency, interact with nature contact to further clarify their joint regulatory mechanisms.

### 5.4 Limitations and suggestions

Although this study provides valuable insights into the relationships among green space exposure, mindfulness, physical activity, and perceived stress, several limitations should be acknowledged.

First, the study employed a cross-sectional survey design with data collected at a single time point, which restricts the ability to make causal inferences. Future research should consider longitudinal or experimental approaches to validate the causal pathways proposed in the model.

Second, the generalizability of our findings is limited by the sampling strategy. Because purposive and convenience sampling was used (e.g., recruiting participants in community parks and WeChat groups), the sample may not fully represent the broader urban population. In addition, the study focused mainly on urban residents, which may not reflect the experiences of rural communities, adolescents, or older adults. Moreover, responses to green spaces may vary by demographic characteristics such as age and gender, which may further limit the generalizability of the dual-path mechanism identified in this study. Future research should consider employing random or stratified sampling, expanding to more diverse populations, and examining subgroup differences to enhance representativeness.

Third, all variables were measured through self-reported questionnaires, which may be subject to biases such as social desirability and recall errors. In particular, participants’ self-assessments of physical activity and green space exposure may not accurately reflect actual behavior or environmental contact. Future studies could incorporate objective measures such as wearable devices, GPS tracking, and GIS-based environmental assessments to enhance data validity.

Finally, the assessment of green space exposure was relatively simplified, relying mainly on subjective perceptions. This limits the ability to examine which types or characteristics of green spaces are most beneficial. Future research should integrate more detailed spatial data—including green space type, area, and accessibility—to provide a more nuanced understanding of how specific environmental features influence mental health outcomes.

## 6 Conclusion

This study developed a moderated mediation model to examine the relationships among green space exposure, mindfulness, physical activity, and perceived stress. The results demonstrate that green space exposure not only directly reduces perceived stress but also indirectly alleviates it by enhancing mindfulness. Moreover, physical activity significantly moderates both the relationship between green space exposure and mindfulness, and the relationship between green space exposure and perceived stress. These findings contribute to a deeper understanding of how natural environments promote mental well-being and offer theoretical support for green urban planning and individualized health interventions.

## Data Availability

The original contributions presented in the study are included in the article/supplementary material, further inquiries can be directed to the corresponding author.
